# Breast papillary lesions diagnosed and treated using ultrasound-guided vacuum-assisted excision

**DOI:** 10.1186/s12893-020-00869-7

**Published:** 2020-09-15

**Authors:** Xiaohui Li, Hua Gao, Minling Xu, Yang Wu, Dezong Gao

**Affiliations:** 1 Nosocomial Infection Department, The Second Hospital, Shandong University Cheeloo College of Medicine, Jinan, 250033 China; 2 Department of Thoracic and General Surgery, Shandong Provincial Chest Hospital, Jinan, 250101 China; 3 Maternity and Child Care Center of DeZhou, DeZhou, 253015 China; 4 Department of Breast Surgery, The Second Hospital, Shandong University Cheeloo College of Medicine, No. 247, Beiyuandajie Street, Jinan, 250033 China

**Keywords:** Papillary lesions, Breast, VAE, Treatment

## Abstract

**Background:**

The management of papillary lesions of the breast remains controversial, and thus, we assessed the value of vacuum-assisted excision (VAE)-guided ultrasound in the diagnosis and treatment of breast papillary lesions.

**Methods:**

We retrospectively reviewed the data of 108 patients with papillary lesions diagnosed using VAE between August 2014 and January 2019. Cases without postoperative breast imaging in the follow-up were excluded, and 85 cases were eligible for the study. The follow-up period ranged from 6 to 53 months, with 38 months on average. All the papillary lesions were located away from the skin or nipple with a size less than or equal to 30 mm, and the lesions categorized as C2-4b were completely excised using VAE. All VAEs were performed using an 8-gauge vacuum-assisted biopsy needle under the guidance of ultrasound using a 10 MHz linear probe.

**Results:**

Most patients with breast papillary lesions were asymptomatic (56.5%), and when the size of the breast papillary lesion was more than 20 mm on ultrasound imaging, atypical hyperplasia may have been concomitant. Breast lesions might have been pathologically diagnosed as papilloma after biopsy when they were categorized as BI-RADS 4a on ultrasound images. The rate of underestimation was 7.7% in papillary lesions diagnosed with VAE, and the recurrence rate of papilloma after VAE was low.

**Conclusions:**

Breast papilloma was a common lesion on ultrasonographic screening, and VAE was applicable for completely excising small papillomas, even papillomas with atypical hyperplasia, to obtain an accurate diagnosis with a low rate of underestimation and recurrence. We believe that papilloma diagnosed by VAE might not require immediate excision, and imaging follow-up may be safe for at least 3 years.

## Background

With the development of screening techniques and imaging diagnostics, more breast papillary lesions are detected in the preclinical stage. Papillary lesions are common in breast biopsies, and the incidence has increased steadily over the past decade [1]. The papillary lesions include benign papilloma, atypical hyperplasia and papillary carcinoma; therefore, it is important to determine whether a lesion is benign or malignant. Core-needle biopsy (CNB) has become widely used in diagnosing breast disease. Some studies have shown that intraductal papilloma (IDP) without atypia diagnosed by core-needle biopsy has a low rate of canceration but that a papilloma with atypia has an increased risk of developing malignancy [2, 3].

Compared with open surgical biopsy, examination by CNB is more easily accepted by patients. However, because of the small portion of lesions with CNB, it is sometimes difficult for pathologists to differentiate between benign and malignant disease [4]. Furthermore, the histological distinction between benign hyperplasia and atypical hyperplasia (AHP) can be subtle, and papillary lesions on CNB are often diagnostically challenging for pathologists. Thus, an adequate number of specimens is essential for a correct diagnosis.

Vacuum-assisted core biopsy (VACB), as a minimally invasive diagnostic method, could provide sufficient samples from a single insertion compared to a traditional core needle biopsy [5]. VACB eliminated the disadvantages of CNB to a large extent and was used to diagnose breast focal lesions, with 98–100% diagnostic accuracy for breast lumps [5]. VACB is primarily used as a diagnostic method; in some cases, especially with smaller lesions, the method can be used therapeutically (vacuum-assisted excision VAE). It was reported that 92% of benign breast lesions with diameters less than 15 mm could be totally removed with VAE [6]. Furthermore, VAE may be used for further diagnoses of benign IDP without atypia on CNB [7].

Considering these advantages of VAE and the dilemmas in dealing with breast papillary lesions, in the present study, we assessed the value of ultrasound-guided VAE in the diagnosis and treatment of breast papillary lesions without initial CNB.

## Material and methods

We retrospectively reviewed the data of 108 patients with papillary lesions diagnosed using VAE between August 2014 and January 2019 at the Department of Breast Surgery, the Second Hospital of Shandong University Cheeloo College of Medicine, China. All breast lesions were categorized as BI-RADS 2 to 4c on ultrasound images or mammograms. We selected cases of lesions that were located away from the skin or nipple with a size less than or equal to 30 mm (peripheral lesions), and lesions that were not categorized as 4c on images were completely excised using VAE. All women who were enrolled in the study underwent an ultrasonographic examination, and those over 40 years of age also underwent mammography. We excluded 5 patients diagnosed with malignant papillary lesions on VAE, and patients without postoperative breast imaging in the follow-up were also excluded; consequently, 85 patients were eligible for inclusion in the study. Of 85 patients, 11 had bilateral lesions, and 96 VAEs were completed. All VAEs were performed using an 8-gauge vacuum-assisted biopsy needle (Mammotome®; Devicor Medical Products, Cincinnati, USA) under ultrasound guidance (LOGIQ 5) using a 10 MHz linear probe.

The average patient age was 43.6 years (26–82 years). Of 85 patients, 48 were asymptomatic (56.5%) with only an abnormal imaging check-up, 22 presented with a discharge, 10 presented with a lump, and only 5 patients complained of breast pain. All patients presented with ultrasonographic nodules, and the size of the biopsied lesions ranged from 5 mm to 30 mm (mean: 12 mm). IDP in the bilateral breast and multifocal papilloma in the unilateral breast were unusual. BI-RADS 4a was the most common category in the prebiopsy image (54.2%). The clinical and morphological features of the biopsied breast lesions are shown in Table 1.

**Table 1:** Baseline characteristics (*n* = 85)

Characteristic	No. (%)
Age^a^ (yr)	43.6 (26–82)
Cause of detection	
Medical checkup without symptom	48 (56.5)
Nipple discharge	22 (25.9)
Palpable mass	10 (11.8)
Breast pain	5 (5.8)
Side	
Right	38 (44.7)
Left	36 (42.4)
Both side	11 (12.9)
IDP with other benign lesions	
Yes	6 (7.1)
No	79 (92.9)
Multifocality	
Yes	10 (11.8)
No	75 (88.2)
BI-RADS category	
C2	9 (10.6)
C3	20 (23.5)
C4a	46 (54.2)
C4b	7 (8.2)
C4c	3 (3.5)
Ultrasonographic identification of intraductal lesion	
Yes	27 (31.8)
No	58 (68.2)
Size (mm)^a^	12 (5–30)
No of samples biopsied^a^	8 (5–28)
IDP with AHP^b^	
Yes	15 (15.6)
No	81 (84.4)

*BI-RADS* Breast imaging reporting and data system, *VAE* Vacuum-assisted excision

^a^ Mean (range). ^b^ 96 VAE including 11 bilateral papillary lesions

Benign IDPs included benign proliferative lesions, such as usual ductal hyperplasia or adenosis, fibrocystic changes, and columnar cell changes. IDP with AHP included lobular intraepithelial neoplasia and ductal atypical hyperplasia. Malignant lesions included papillary cancer with or without ducal carcinoma in situ (DCIS). Two pathologists with extensive expertise in breast lesions reviewed all cases. The patients were followed up with clinical and ultrasound examinations for 3 and 6 months after the operation and then with ultrasound and mammography (at ages greater than 40 years) annually. The follow-up period ranged from 6 to 53 months, with 38 months on average. This study was approved by the Ethics Committee of the Second Hospital of Shandong University, and informed consent was waived.

### Statistical analysis

Statistical analysis was performed using SPSS version 21.0 software (IBM Corp, Armonk, USA). A t-test was used to compare the mean diameters between IDP lesions and IDP with APH lesions. The chi-square test was used to compare the ultrasound identification efficacy between patients with nipple discharge and patients without nipple discharge. Statistical significance was defined as a *p*-value < 0.05. All reported *p*-values were two-sided.

## Results

Of the 96 VAEs, the initial diagnoses were 81 IDPs and 15 IDPs with AHP. The mean lesion diameter in IDPs with AHP was significantly longer than that in IDPs (mean 21 mm in IDP with AHP and 13 mm in IDP, Table 2). Except for lesion diameter, IDP with AHP had no special characteristics compared with IDP. The rate of ultrasonographic identification of IDP in patients with nipple discharge was higher than that in patients without nipple discharge (Table 3). To verify the diagnostic accuracy of VAE, 26 patients (10 patients with BI-RADS category 4b and 4c on images) underwent open extended excision after VAE (including 16 IDPs and 10 IDPs with AHP). The results of all pathological examinations were the same as the initial diagnosis except for 2 cases. One was diagnosed as IDP with AHP initially diagnosed as IDP, and the other was diagnosed as intraductal papillary carcinoma initially diagnosed as IDP with sclerosing adenosis. The BI-RADS category was 4a on US image in the upgraded malignant patient, but her contralateral mass was invasive ductal carcinoma. However, the histopathological examinations were all benign in 10 patients with BI-RADS category 4b and 4c on images (IDP in 6 patients and IDP with APH in 4 patients). Open excision revealed an underestimation of 2/26 (7.7%). All 26 patients who underwent open surgery were followed regularly, and no suspicious alteration was found on imaging during the follow-up period.

**Table 2:** Diameters of IDP lesions and IDP with APH lesions

Lesions	Diameter (mm)	s	*P*
	mean (range)		
IDP	13 (5–30)	8.7	
IDP with APH	21 (12–28)	12.4	<0.01

**Table 3:** US identification of IDP in 96 VAEs with nipple discharge or not

US identification	Nipple discharge		X^2^	*P*
	Yes	No		
Yes	14	17		
No	8	57	12.83	<0.01

*US* Ultra-sonogram

After VAE, 59 patients without open excision underwent regular follow-up. Five (8.5%) of the 59 patients presented with hypoechoic lesions in the primary site (5–11 mm) during the follow-up period, and 2 patients underwent VAE again. The histopathologic diagnoses were IDP and adenosis, respectively. Four patients (6.8%) had new nodules less than 5 mm in diameter but not in primary sites, and the US categories were C2 and C3. These four patients did not undergo VAE, and no enlargement process was revealed in the follow-up. In 5 patients with multifocal lesions, nodules less than 5 mm in diameter were not excised in the initial VAE. Three patients presented no nodules, and no alterations were found in the nodules of the other 2 patients during the follow-up period (Table 4).

**Table 4:** The follow-up results of patients underwent no open surgery (*n* = 59)

Results	No. (%)
New nodules in primary site	5 (8.5)
Re-operation	2 (40)
New nodules not in primary site	4 (6.8)
Multifocal nodules not excised	5 (8.5)
No alteration	2
No nodule	3

## Discussion

Papillary lesions are a heterogeneous group of breast lesions that include benign papillomas, atypical papillomas, and papillary carcinomas. Intraductal papilloma usually presents with nipple discharge or a palpable mass, and surgical management is the common procedure. However, an increasing number of papillomas are occasionally detected by image screening in the preclinical stage, which usually presents with no clinical symptoms. Some of these lesions are seen sonographically as solid nodules that are not distinguishable from other solid lesions in the breast. It has been suggested that these preclinical papillary lesions require additional surgical excision because they tend to cancerate [8]. In the present study, we found that 56.5% of patients with papilloma were asymptomatic, and 54.2% were categorized as BI-RADS 4a on ultrasonographic images. Only 32.3% of intraductal lesions were identified on an ultrasonographic check-up. However, patients with nipple discharge were more easily identified with ultrasonography (63.6%), possibly because the papilloma was visible in the dilated duct, which was full of fluids. The average papilloma size was 12 mm (5–30 mm) in our study, so it was difficult to differentiate small, impalpable papillary lesions. It was especially difficult to excise lesions with such a small size, but CNB guided by ultrasound was the applicable method to diagnose these lesions. However, because of insufficient tissue collected at biopsy, it was sometimes difficult to distinguish malignant from benign papillary lesions using CNB [9].

Some studies have recommended that papillary lesions diagnosed by CNB, especially those with atypia, should be subjected to open surgery for an accurate diagnosis because the rate of upgrading to malignancy is high in such situations [10, 11]. Regarding benign IDP without atypia, previous studies have reported that the rates of upgrading vary widely following excision. Some studies have shown that IDPs are significantly associated with higher-grade lesions, and open excision is recommended in all cases [10, 12]. However, other reports have suggested clinical and imaging follow-up rather than surgical excision because the rate of upgrading is low in IDP without atypia [13, 14]. Therefore, the management of benign IDPs diagnosed by CNB remains controversial. Several studies have shown that the upgrading rate after open excision is associated with the adequacy of samples in biopsy lesions, even though the needle gauge used in CNB plays an important role in upgrading the rate of IDP [15, 16]. In our study, we found an underestimation of 2/26 (7.7%) following open excision after VAE. IDP with AHP was diagnosed in one patient initially diagnosed with IDP on VAE, and the other was diagnosed with intraductal papillary carcinoma after an initial diagnosis of IDP with sclerosing adenosis on VAE. Our underestimation rate was lower than those of other authors. For example, Tatarian et al. found that 21.3% of patients who were initially diagnosed with benign papilloma with CNB had IDP with atypia following surgical excision, and the majority of the atypical lesions were obtained from the tissue surrounding the papilloma [17]. The reason for the low rate of underestimation in our patients may be the sufficient samples (mean 8 tissue cores) obtained with VAE (8 gauge needle). Cassano et al. [18] also believed that further verification with open excision was not necessary in patients diagnosed with benign lesions by VAE. They showed that patients diagnosed with IDP with VAE exhibited no recurrence or progression when followed up for several months via imaging check-ups [18]. Obviously, the several-month follow-up was too short in the above study; however, a follow-up was conducted for 38 months in our patients who underwent VAE, and suspicious alterations were not found on images, which means VAE is an applicable method of diagnosing and treating breast papillary lesions. However, of the two downregulated patients, one was diagnosed with intraductal papillary cancer after open surgery. This patient suffered simultaneously from contralateral invasive ductal cancer, and her initial diagnosis was IDP with sclerosing adenosis on VAE. Consequently, attention should be paid to such patients when VAE is applied.

In the present study, we found that the average lesion diameter in IDPs with AHP (21 mm) was greater than that in IDPs (13 mm), which means that AHP was associated with lesion size. It has been reported that the BI-RADS category is associated with upgrade rates in benign IDP diagnosed by CNB [19]. In our study, 10 patients with BI-RADS category 4b and 4c on US images underwent open surgery. The histopathological examinations were IDPs in 6 patients and IDP with APH in 4 patients. We could not find the relation between the upgrading rate and BI-RADS category, which might be because of fewer upgrading cases. However, we found that 54.2% of papillary lesions were categorized as BI-RADS 4a on images, and the C3, and C4b, C4c category only accounted for 11.7% (10 cases) of the total patients. Therefore, papillary lesions must be kept in mind when breast lesions are categorized as 4a on ultrasonographic images.

Several reports have revealed that in more than 95% of patients, ultrasound-guided VAE can entirely remove papillomas that measure less than 15 mm and therefore has therapeutic value to avoid open surgery [7, 20]. In our patients, the largest diameter of the lesion was 30 mm, and we completely excised the lesions via VAE guided by ultrasonography. In the follow-up period, 8.5% (5/59) of patients presented with hypoechoic lesions in the primary site on ultrasonogram, 2 patients underwent VAE again, and the histopathological diagnoses were IDP and adenosis. The other 3 patients were followed up, and no suspicious alterations were found on images. Because only two patients underwent VAE again, the recurrence rate could not be determined. We might not have entirely removed all lesions with VAE in these patients because the lesion sizes were larger than the needle groove, and tiny residual lesions could not be detected with ultrasonography. There were 5 cases of multifocal lesions on the initial ultrasonogram in our study, and lesions less than 5 mm in diameter were not excised. After an average follow-up of 38 months, we found that 3 in 5 patients presented no lesions, and no enlargement process was found in the other 2 patients’ lesions. Donaldson et al. [21] found that the 7-year cumulative breast cancer incidence rate was only 10% in patients who had an initial diagnosis of AHP on CNB, so they believed that close imaging follow-up was applicable to these patients. Furthermore, breast papillary carcinoma is rare, representing only 1–2% of all breast malignancies [22]. Therefore, we consider that it is not necessary to excise lesions less than 5 mm in diameter, especially in patients with multifocal papillomas.

## Conclusions

VAE was applicable for completely excising papillomas, even IDPs with AHP, for an accurate diagnosis because of sufficient samples for pathological examination. In addition to the lower underestimation rate, the recurrence rate was also very low in IDP diagnosed via VAE, so VAE was applicable for excising completely small lesions. Because of the short period of follow-up (approximately 3 years), the canceration rate of papilloma after VAE deserves further study.

## Data Availability

The datasets used and/or analysed during the current study are available from the corresponding author on reasonable request.

## Abbreviations

CNB: Core needle biopsy
VAE: Vacuum-assisted excision
IDP: Intraductal papilloma
AHP: Atypical hyperplasia
BI-RADS: Breast image-report and data system
